# A novel anti-TNF-α drug ozoralizumab rapidly distributes to inflamed joint tissues in a mouse model of collagen induced arthritis

**DOI:** 10.1038/s41598-022-23152-6

**Published:** 2022-10-27

**Authors:** Shohei Oyama, Kosuke Ebina, Yuki Etani, Makoto Hirao, Masanao Kyuuma, Yasuyuki Fujii, Katsuya Iwata, Bunichiro Ogawa, Tomoya Hasegawa, Sasagu Kawano, Yutaka Nakanishi, Seiji Okada, Ken Nakata

**Affiliations:** 1 Department of Musculoskeletal Regenerative Medicine, Osaka University Graduate School of Medicine, Osaka, Japan; 2 Department of Orthopaedic Surgery, Osaka University Graduate School of Medicine, Osaka, Japan; 3 Taisho Pharmaceutical Co., Ltd., Saitama, Japan; 4 Department of Medicine for Sports and Performing Arts, Osaka University Graduate School of Medicine, Osaka, Japan

**Keywords:** Rheumatic diseases, Antibody therapy

## Abstract

In clinical studies, the next-generation anti-tumor necrosis factor-alpha (TNF-α) single domain antibody ozoralizumab showed high clinical efficacy shortly after the subcutaneous injection. To elucidate the mechanism underlying the rapid onset of the effects of ozoralizumab, we compared the biodistribution kinetics of ozoralizumab and adalimumab after subcutaneous injection in an animal model of arthritis. Alexa Fluor 680-labeled ozoralizumab and adalimumab were administered by subcutaneous injection once (2 mg/kg) at five weeks after induction of collagen-induced arthritis (CIA) in an animal arthritis model. The time-course of changes in the fluorescence intensities of the two compounds in the paws and serum were evaluated. The paws of the CIA mice were harvested at four and eight hours after the injection for fluorescence microscopy. Biofluorescence imaging revealed better distribution of ozoralizumab to the joint tissues than of adalimumab, as early as at four hours after the injection. Fluorescence microscopy revealed a greater fluorescence intensity of ozoralizumab in the joint tissues than that of adalimumab at eight hours after the injection. Ozoralizumab showed a significantly higher absorption rate constant as compared with adalimumab. These results indicate that ozoralizumab enters the systemic circulation more rapidly and is distributed to the target tissues earlier and at higher levels than conventional IgG antibodies. Our investigation provides new insight into the mechanism underlying the rapid onset of the effects of ozoralizumab in clinical practice.

## Introduction

Rheumatoid arthritis (RA) is a systemic, chronic, autoimmune inflammatory disease. The target tissue is mainly synovial joints, and the disease is characterized by synovial inflammation, swelling, cartilage destruction, and bone erosion. Long-lasting damage to the joint tissues finally lead to disability^1–3^. Tumor necrosis factor-alpha (TNF-α) is known to be one of the most important mediators associated with the pathogenesis of RA. TNF-α is mainly secreted by monocytes and macrophages, locally interacts with TNF receptors in an autocrine or paracrine manner, and triggers a pro-inflammatory cascade^4^. Anti-TNF-α therapies have shown high therapeutic efficacy against RA, and the following five drugs are currently available: infliximab, etanercept, adalimumab, golimumab, and certolizumab^3,5–7^. Among them, adalimumab, a recombinant fully humanized anti-TNF-α monoclonal IgG antibody with a molecular weight of approximately 150 kDa, is the most commonly used anti-TNF-α biologic, accounting for 47% of all users in the United States^8,9^.

**Figure 1:**
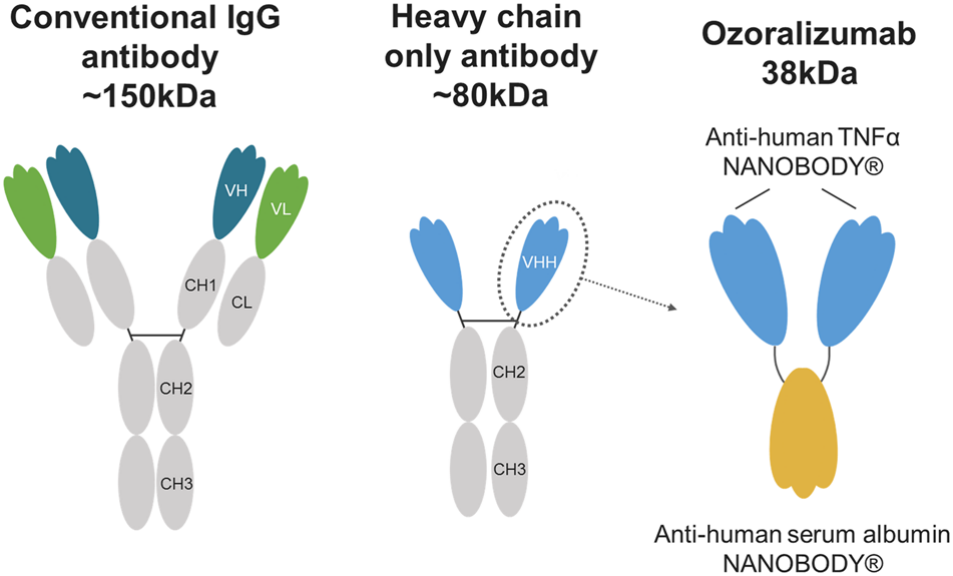
Schematic representation of a conventional IgG antibody, heavy-chain-only antibody, and ozoralizumab.

The next-generation anti-TNF-α single domain antibody ozoralizumab is a recombinant humanized trivalent NANOBODY® compound consisting of two anti-TNF-α NANOBODY® (variable region domain of heavy-chain antibodies) molecules and one anti-human serum albumin (HSA) NANOBODY® molecule^10^ (Fig. 1). It has a molecular weight of 38 kDa, which is about one quarter the size of representative existing anti-TNF-α IgG antibodies. The HSA-binding domain contributes to the long serum half-life of ozoralizumab^11^. In a human clinical trial, subcutaneous injection of 30 mg of ozoralizumab with MTX improved the disease activity (DAS28-CRP, Pt-GA) as early as at three days after the injection^12^, while administration of conventional IgG antibodies, including adalimumab, improved the disease activity as early as at two weeks after administration^13–15^, which suggests that ozoralizumab may have a more rapid onset of action. Since a rapidity of onset of action is determined by the time at which the concentration reaches the therapeutic level at the target site, the concentration of ozoralizumab in the inflamed joints is assumed to increase more rapidly. However, no published study has been conducted to compare the biodistribution of NANOBODY® molecules and conventional IgG antibodies in animal models of RA, and the underlying mechanism has not yet been clearly revealed.

Drugs administered into the subcutaneous interstitium are taken up through the subcutaneous capillaries or lymphatic vessels to enter the systemic circulation, to then become distributed to the peripheral tissues. This entire process is intricately regulated both by the properties of the injected molecules and by host factors^16–18^. Molecular size is one of the most important factors influencing the biodistribution kinetics after subcutaneous injection, and compounds with high molecular weights, including IgG antibodies, are absorbed more slowly than compounds with lower molecular weights^16,19^. In addition, the interactions between compounds and specific biological molecules contributes to the tissue retention. For example, proteins fused with anti-serum albumin antibodies are known to accumulate in the inflamed joints^20,21^. Based on these reports, we hypothesized that ozoralizumab, due to its smaller molecular weight and serum albumin binding ability, would show more rapid and better distribution in the inflamed tissues as compared with conventional IgG antibodies; this could explain the mechanism underlying the rapid onset of action of ozoralizumab observed in clinical studies.

Therefore, the objectives of this study were to investigate the biodistribution of ozoralizumab in comparison to that of adalimumab, an anti-TNF-α IgG antibody, in a mouse model of collagen-induced arthritis (CIA mouse model), which is a well-established model that mimics human RA^22^, and to uncover the mechanism underlying the rapid onset of action of ozoralizumab in clinical practice. Towards these goals, we used an in vivo imaging system (IVIS), histopathological examination, and pharmacokinetic evaluations after subcutaneous injection of the fluorescence-labeled antibodies.

## Results

### Labeling of ozoralizumab and adalimumab with Alexa Fluor® 680

Ozoralizumab and adalimumab were labeled with Alexa 680 via the N-terminal amino acid and lysine residues at an average dye/protein molar ratio of 1.6–1.7, to obtain Alexa 680-labeled ozoralizumab (ozoralizumab-Alexa 680) and Alexa 680-labeled adalimumab (adalimumab-Alexa 680). We then investigated the effects of the labeling on the binding affinity of ozoralizumab for mouse serum albumin (MSA). The dissociation constants (K_d_) between ozoralizumab or ozoralizumab-Alexa 680 and MSA were 4.0 × 10^–7^ (M) and 9.5 × 10^–7^ (M), respectively (Supplementary Table 1). These results indicated that labeling with Alexa 680 exerted limited effect on the binding affinity of ozoralizumab for MSA.

### Biodistribution of ozoralizumab-Alexa 680 and adalimumab-Alexa 680 after a single subcutaneous injection analyzed using an in vivo imaging system

**Figure 2:**
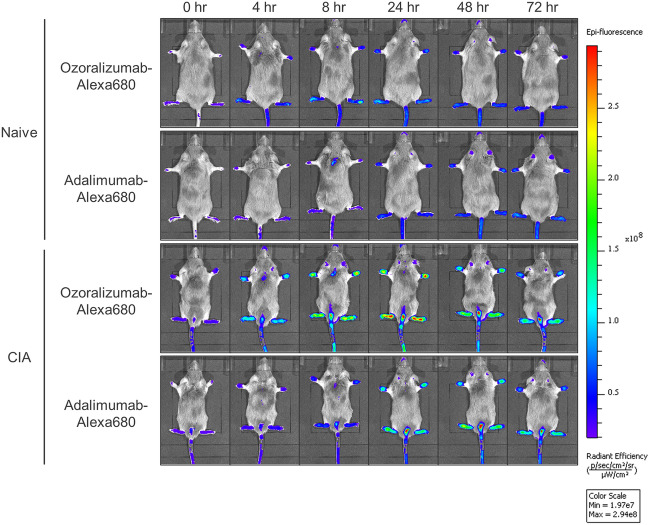
Biofluorescence IVIS images after a single subcutaneous injection of ozoralizumab-Alexa 680 or adalimumab-Alexa 680. Before and at 4, 8, 24, 48, and 72 h after subcutaneous injection of 2 mg/kg ozoralizumab-Alexa 680 or 2 mg/kg adalimumab-Alexa 680, biofluorescence IVIS images of the whole body were obtained. The fluorescence intensities are represented on a false color scale, with blue indicating low intensities and red indicating high light intensities.

**Figure 3:**
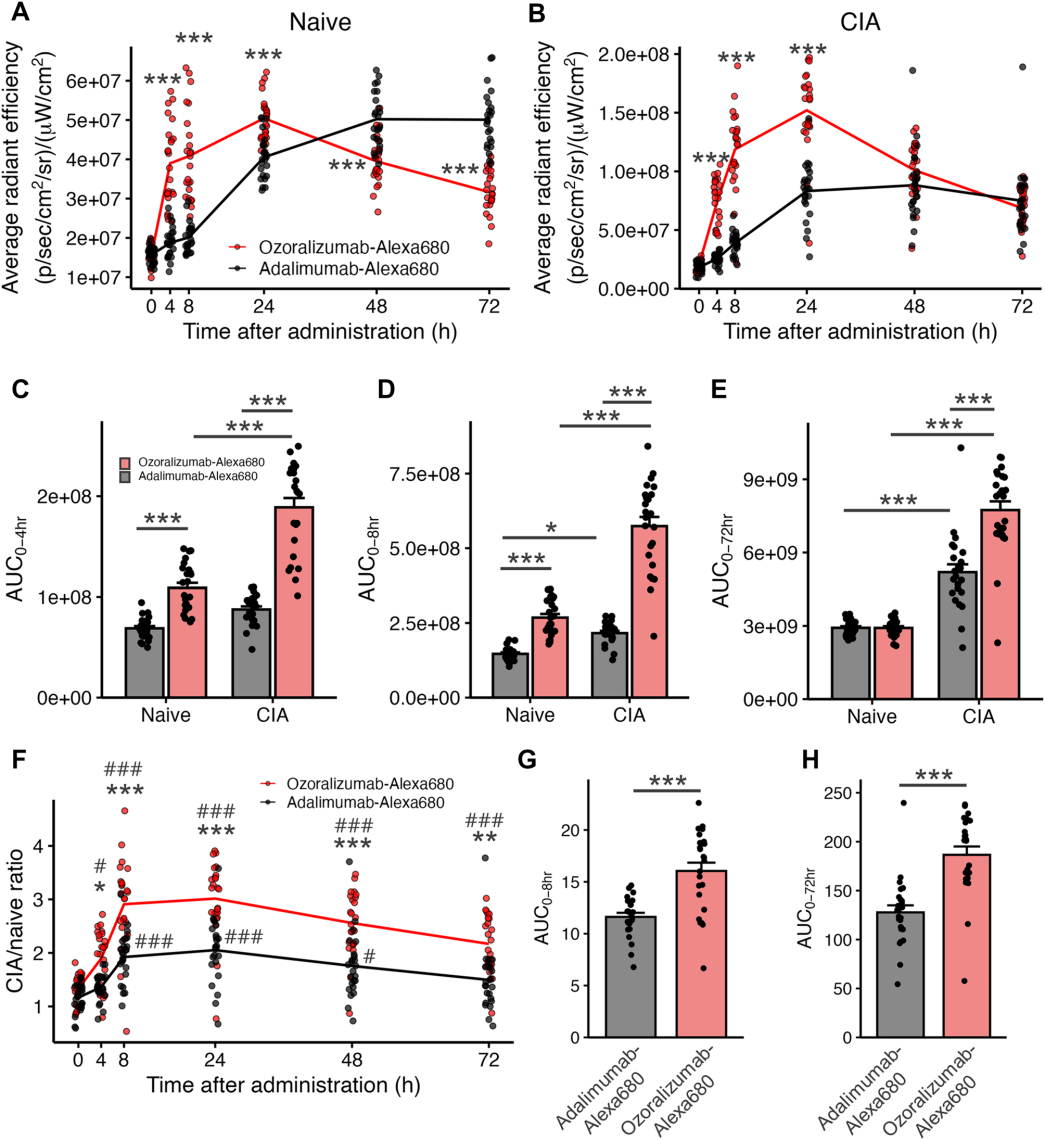
Time-course analysis of in vivo biofluorescence images. Quantification of the average radiant efficiency in the paws of naive mice (**A**) and CIA mice (**B**). *P* values were calculated by two-way ANOVA with post-hoc Tukey’s test. Area under the curve (AUC) of the average radiant efficiency from 0 to 4 h (**C**), 0 to 8 h (**D**), and 0 to 72 h (**E**). *P* values were calculated by two-way ANOVA with post-hoc Tukey’s test. (**F**) The ratio of the average radiant efficiency in the CIA model mice to the average radiant efficiency in naive mice over time. P values were calculated by two-way ANOVA with post-hoc Tukey’s test. AUC of the ratio of the average radiant efficiency in the CIA model mice to the average radiant efficiency in naive mice from 0 to 8 h (**G**) and 0 to 72 h (**H**). *P* values were calculated using Welch’s t-test. Dot plots represent each individual data, lines represent the means, and bars represent the means ± SEM. red: ozoralizumab-Alexa 680; black: adalimumab-Alexa 680. n = 24 paws (6 mice) per group. *P < 0.05, **P < 0.01, ***P < 0.001. #*P* < 0.05, ###*P* < 0.001, vs. 0 h.

To investigate the biodistribution of ozoralizumab and adalimumab in the joint tissues, biofluorescence images of the antibodies were obtained by IVIS before, and at 4, 8, 24, 48, and 72 h after a single subcutaneous injection of ozoralizumab-Alexa 680 or adalimumab-Alexa 680 in naive mice and CIA model mice (Fig. 2). The fluorescence intensities were represented on a false color scale, with blue indicating low, and red indicating high light intensity. Fluorescence was detected in the paws and tail, and to some degree, in the ear. Using the ROI tool, the fluorescence intensity in each paw was quantified as the average radiant efficiency at each time-point. There were significant effects on the average radiant efficiency of injection of Alexa 680-labeled antibodies in both the naive mice (Fig. 3A, F1, 276 value = 22.06, *P* < 0.001) and CIA mice (Fig. 3B, F1, 276 value = 163.11, *P* < 0.001). The average radiant efficiency in the ozoralizumab-Alexa 680-injected naive mice was significantly higher than that in the adalimumab-Alexa 680-injected naive mice at 4, 8, and 24 h (*P* < 0.001). Similar to the case in the naive mice, in the CIA mice, the average radiant efficiency in the ozoralizumab-Alexa 680 group was significantly higher than that in the adalimumab-Alexa 680 group at 4, 8, and 24 h (*P* < 0.001). Moreover, the maximal fluorescence intensity in the paws of the ozoralizumab-Alexa 680 group was observed at 24 h, while that in the adalimumab-Alexa 680 group was observed at 48–72 h. To compare the accumulation of Alexa 680-labeled compounds in the joints, the AUC was calculated from 0 to 4, 8, and 72 h. In both ozoralizumab-Alexa 680-injected naive and CIA mice, the AUC_0–4 h_ and AUC_0–8 h_ were significantly higher as compared with those in the adalimumab-Alexa 680 mice (Fig. 3C and D, *P*_naive, 0–4 h_ < 0.001, *P*_CIA, 0–4 h_ < 0.001, *P*_naive, 0–8 h_ < 0.001, *P*_CIA, 0–8 h_ < 0.001). In the CIA mice, the AUC_0–72 h_ in the ozoralizumab group was significantly higher than that in the adalimumab group (Fig. 3E, *P* < 0.001). These results indicate that ozoralizumab was distributed more rapidly and at higher levels in the joint tissues than adalimumab. To assess the distribution of ozoralizumab and adalimumab under inflammatory conditions, the CIA/naive fluorescence ratio was calculated (Fig. 3F). The CIA/naive fluorescence ratio was significantly increased from the baseline at 4, 8, 24, 48, and 72 h after injection of ozoralizumab-Alexa 680 (*P*_4hr_ = 0.022, *P*_8hr_ < 0.001, *P*_24hr_ < 0.001, *P*_48hr_ < 0.001, *P*_72hr_ < 0.001), and at 8, 24, and 48 h after the injection of adalimumab-Alexa 680 (*P*_8hr_ < 0.001, *P*_24hr_ < 0.001, *P*_48hr_ = 0.012), which indicated that more extensive distribution of both ozoralizumab-Alexa 680 and adalimumab-Alexa 680 was observed in the paws of the CIA mice as compared with that in the paws of the naive mice. Note that injection of Alexa 680-labeled antibodies exerted a significant effect on the CIA/naive fluorescence ratio (F_1, 276_ = 112.4, *P* < 0.001), which was significantly higher in the ozoralizumab-Alexa 680 group than in the adalimumab-Alexa 680 group as early as at four hours after the injection (*P*_4hr_ = 0.042, *P*_8hr_ < 0.001, *P*_24hr_ < 0.001, *P*_48hr_ < 0.001, *P*_72hr_ = 0.0018). The AUC_0–8 h_ and AUC_0–72 h_ of the fluorescence ratio-time profiles were significantly higher in the ozoralizumab-Alexa 680 group than in the adalimumab-Alexa 680 group (Fig. 3G,H, *P* < 0.001). Taken together, large amounts of ozoralizumab-Alexa 680 and adalimumab-Alexa 680 entered the joint tissues under pathological conditions as compared with the healthy condition, and the rate of increase was higher in the ozoralizumab-Alexa 680 group from as early as four hours after the injection.

### Fluorescence microscopy of the paws of the CIA mice after administration of ozoralizumab-Alexa 680 and adalimumab-Alexa 680

**Figure 4:**
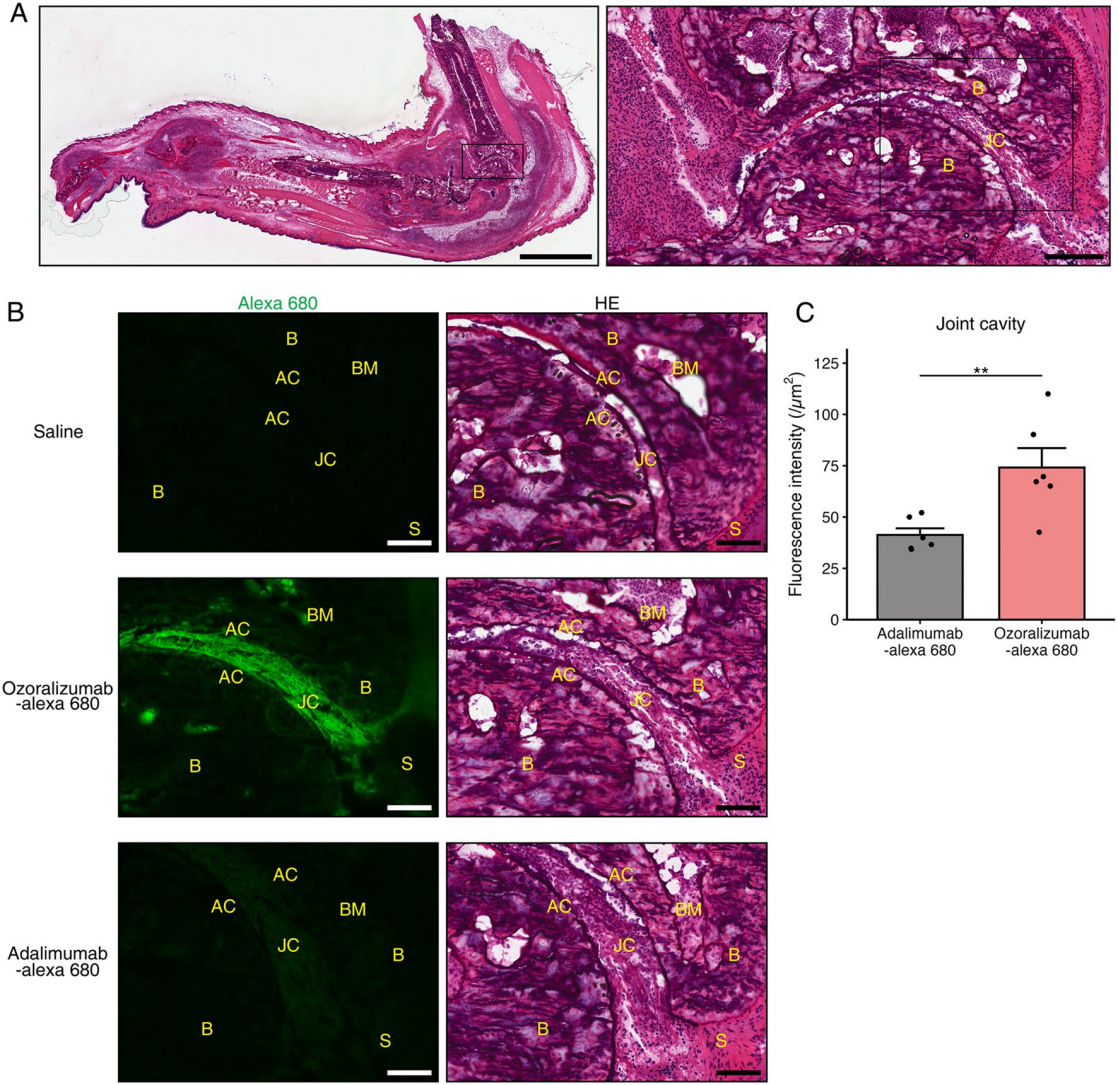
Fluorescence microscopy and morphometry of the talocrural joints at eight hours after a single subcutaneous injection. (**A**) Paws of the CIA mice were sectioned in the sagittal plane and stained with hematoxylin & eosin (HE). Scale bar: 2 mm (left), 200 µm (right). B: bone; JC: joint cavity. (**B**) Fluorescence microscopy of the talocrural joints eight hours after a single subcutaneous injection of ozoralizumab-Alexa 680 and adalimumab-Alexa 680. The green fluorescence represents the administered compounds labeled with Alexa 680 (left). Serial sections were stained with HE (right). Scale bar: 100 µm. B: bone; BM: bone marrow; AC: articular cartilage; JC: joint cavity; S: synovium. (**C**) The fluorescence value of Alexa 680 in the joint cavity at eight hours after subcutaneous injection was analyzed. Dot plots represent each individual data and bars represent the means ± SEM. *P* values were calculated using the Wilcoxon rank sum test. **P < 0.01.

**Figure 5:**
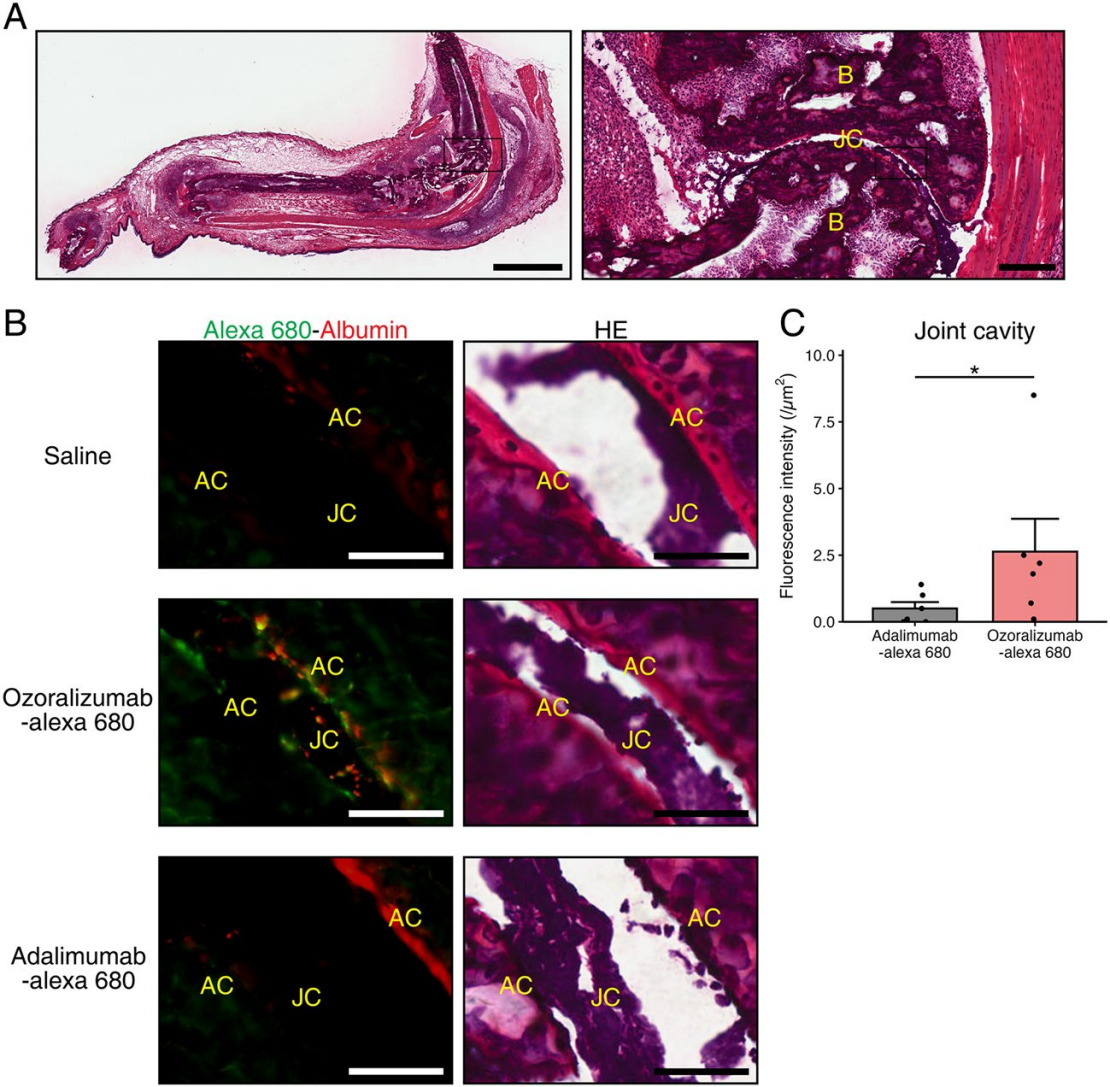
Localization of MSA and test articles in the talocrural joint. (**A**) Paws of the CIA mice were sectioned in the sagittal plane and stained with hematoxylin & eosin (HE). Scale bar: 2 mm (left), 200 µm (right). B: bone; JC: joint cavity. (**B**) At eight hours after a single subcutaneous injection of ozoralizumab-Alexa 680 and adalimumab-Alexa 680, immunohistochemistry was performed on sagittal sections of the talocrural joints using antibody against MSA. The green fluorescence represents the administered compounds labeled with Alexa 680 and red fluorescence represents albumin. Serial sections were stained with HE (right). Scale bar: 50 µm. AC: articular cartilage; JC: joint cavity. (**C**) The fluorescence value of Alexa 680 merged with the fluorescence of Alexa 488-labeled albumin antibody in immunohistochemistry sections of the joint cavity at eight hours after subcutaneous injection was analyzed. Dot plots represent each individual data and bars represent the means ± SEM. *P* values were calculated using the Wilcoxon rank sum test. *P < 0.05.

To assess the biodistribution of ozoralizumab-Alexa 680 and adalimumab-Alexa 680 to inflamed joints, fluorescence microscopy of the paws of the CIA mice was performed at four and eight hours after injection of the labeled antibodies. Fluorescence of ozoralizumab-Alexa 680 was observed in the joint cavity, articular cartilage, synovium, bone marrow, and subcutaneous tissue at both four and eight hours after injection of the antibody, with the fluorescence intensity being more marked at eight hours than that at four hours. Fluorescence of adalimumab-Alexa 680 was observed in the joint cavity, synovium, bone marrow, and subcutaneous tissue, but not in the articular cartilage, at both four and eight hours after injection of the antibody. Although the fluorescence intensity of adalimumab-Alexa 680 at eight hours after the injection was more pronounced than that at four hours, the fluorescence intensity of ozoralizumab-Alexa 680 was much higher than that of adalimumab-Alexa 680 at both four and eight hours after injection (Fig. 4A,B). Morphometry of the fluorescence intensity in the joint cavity revealed that the fluorescent intensity in ozoralizumab-Alexa 680 mice was significantly higher than that in the adalimumab-Alexa 680 mice (Fig. 4C, *P* = 0.0087). In addition, fluorescence immunohistochemistry with Alexa 488-labeled antibodies for MSA and mouse TNF-α in the talocrural joints at eight hours after subcutaneous injection of ozoralizumab-Alexa 680 or adalimumab-Alexa 680 revealed fluorescence derived from ozoralizumab-Alexa 680 was observed near the fluorescence derived from albumin or TNF-α in the joint cavity (Fig. 5A,B, Supplementary Fig. 1A), and the fluorescence intensity of ozoralizumab-Alexa 680 co-localized with that of Alexa 488-labeled antibody against MSA or mouse TNF-α was higher than that of adalimumab-Alexa 680 (Fig. 5C; *P* = 0.048, Supplementary Fig. 1B; *P* = 0.0043). Taking into account all these observations, the results suggest that ozoralizumab was better distributed than adalimumab at the sites of action of arthritis, where TNF-α is abundantly distributed.

### Pharmacokinetics of ozoralizumab-Alexa 680 and adalimumab-Alexa 680 after a single subcutaneous injection

**Figure 6:**
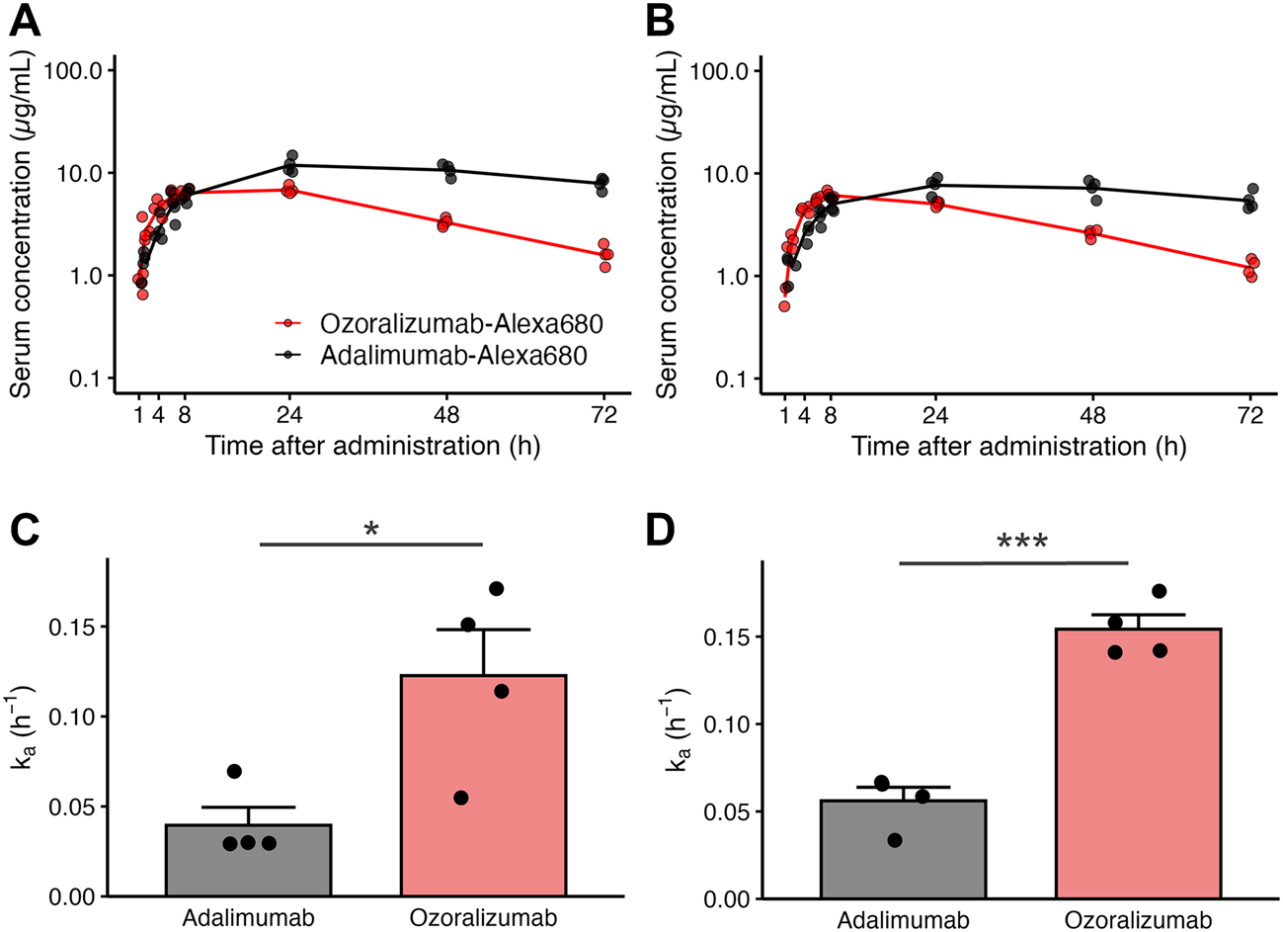
Serum concentration–time profiles of ozoralizumab-Alexa 680 and adalimumab-Alexa 680 after a single subcutaneous injection. At 1, 2, 4, 6, 8, 24, 48, and 72 h after subcutaneous injection of 2 mg/kg of ozoralizumab-Alexa 680 or 2 mg/kg of adalimumab-Alexa 680, the antibody concentrations in the serum were investigated in naive mice (**A**) and CIA model mice (**B**). Dot plots represent each individual data, and the lines represent the mean; red, ozoralizumab-Alexa 680; black, adalimumab-Alexa 680; n = 4 mice per group. The absorption rate constant (k_a_) was analyzed by 1-compartmental analysis in naive mice (**C**) and CIA model mice (**D**). Bars represent the means ± SEM. *P* values were calculated using Welch’s t-test. *P < 0.05, ***P < 0.001.

To examine the relationship between the serum concentrations and biodistribution of ozoralizumab-Alexa 680 and adalimumab-Alexa 680, the serum concentration–time profiles were investigated in the naive (Fig. 6A) and CIA mice (Fig. 6B). After a single subcutaneous injection of ozoralizumab-Alexa 680 at 2 mg/kg to CIA and naive mice, a C_max_ of 6.14 to 6.91 µg/mL was reached at 8 to 16 h with a k_a_ of 0.123 to 0.154 h^−1^and declined with a t_1/2_ of 23 h, and the AUC_0–72 h_ was 251 to 311 h*µg/mL (Supplementary Table 2). After a single subcutaneous injection of adalimumab-Alexa 680 at 2 mg/kg to CIA and naive mice, a C_max_ of 7.75 to 12.2 µg/mL was reached at 24 to 30 h with a k_a_ of 0.0395 to 0.0561 h^−1^, and declined with a t_1/2_ of 92 to 110 h, and the AUC_0–72 h_ was 453 to 660 h*µg/mL (Supplementary Table 2). The k_a_ of ozoralizumab-Alexa 680 was significantly higher than that of adalimumab-Alexa 680 in both the naive mice (Fig. 6C, *P* = 0.04) and CIA mice (Fig. 6D, *P* < 0.001), indicating that the subcutaneously injected ozoralizumab-Alexa 680 was more rapidly absorbed into the blood stream of the mice than subcutaneously injected adalimumab-Alexa 680.

## Discussion

Overall, ozoralizumab was more rapidly absorbed from the subcutaneous interstitium and better distributed to joint tissues with or without inflammation, as compared with the anti-TNF-α IgG antibody, adalimumab. To the best of our knowledge, this is the first preclinical study to compare the biodistribution kinetics of ozoralizumab and adalimumab in a head-to-head manner, and our results contribute to a better understanding of the mechanisms underlying the rapid therapeutic effects of ozoralizumab in clinical practice.

Several clinical studies have reported that subcutaneous injection of adalimumab, golimumab, or etanercept to healthy individuals or RA patients resulted in a C_max_ of several μg/ml^23–26^. In this study, the C_max_ following subcutaneous injection of 2 mg/kg of adalimumab-Alexa 680 or ozoralizumab-Alexa 680 to CIA mice (6.1 µg/ml and 7.8 µg/ml, respectively, Supplementary Table 2) was equivalent to the values found in clinical studies, which suggests that subcutaneous injection of the compounds at 2 mg/kg in this animal study reflected the clinical situation.

Our pharmacokinetic analysis showed that the subcutaneous absorption rate constant (k_a_: hr^- 1^) of ozoralizumab was higher than that adalimumab, regardless of the presence/absence of inflammation (Fig. 6C and D), indicating that ozoralizumab was more rapidly absorbed and reached the systemic circulation. In the present study, we administered approximately four times the dose, on a molar basis, of ozoralizumab-Alexa 680 as compared with that of adalimumab-Alexa 680. However, the difference in the molar dose seems to have little effect on the absorption rate, because the drug dose is an independent determinant of the k_a_. The rate of absorption of subcutaneously injected drugs is highly dependent on the molecular size of the drugs^16,19,27^. Therefore, the higher k_a_ of ozoralizumab is likely to be attributable to its lower molecular weight. In addition, we found that the temporal changes in the fluorescence intensities of ozoralizumab and adalimumab in the paws corresponded to the temporal changes in their serum concentrations. These results suggest that the subcutaneous absorption process is the rate-limiting step in the distribution of ozoralizumab and adalimumab to the target sites.

An increase of the CIA/naive fluorescence ratio as compared with that at the baseline was observed for both ozoralizumab and adalimumab (Fig. 3F), suggesting enhanced distribution of the antibodies into the joint tissues under the inflammatory condition. Under physiological conditions, the blood vessels in the synovium are composed of continuous fenestrated endothelium, which prevents penetration of larger proteins, including the serum albumin (approximately 65 kDa) from the blood stream to the interstitium across the blood vessels^28^. However, under inflammatory conditions, such as RA, capillary angiogenesis and increased vascular permeability occur, and even proteins of high molecular weights can pass through the vascular endothelium to reach inflamed joint tissues^29,30^. Taken together with the lower serum concentration of the antibodies in CIA mice than in naive mice, the higher distribution in the joint tissues in the CIA mice than in naive mice results from the increased permeability of the vascular endothelium caused by inflammation.

We showed that the CIA/naive fluorescence ratio in the paws of ozoralizumab-Alexa 680 mice was significantly higher than that in the adalimumab-Alexa 680 mice (Fig. 3F–H). Considering that the AUC_0–72 h_ in naive mice was comparable between the ozoralizumab-Alexa 680 group and adalimumab-Alexa 680 group (Fig. 3E), ozoralizumab-Alexa 680 was likely to be better distributed to inflamed joint tissues as compared with adalimumab-Alexa 680. A previous study suggested that during inflammation, the tissue distribution of proteins increased due to the higher blood endothelial permeability, and proteins with larger molecular weights were affected to a greater degree than proteins with smaller molecular weights^29^. According to this report, the CIA/naive fluorescence ratio of adalimumab was thought to be higher due to its fourfold higher molecular weight than ozoralizumab, however, the results were contrary. One possible explanation for the higher ratio of ozoralizumab could be its ability to bind to serum albumin. A previous animal study reported that large amounts of serum albumin accumulated in inflamed paws^31^, and the results of the immunohistochemical examination in our study were consistent with that report (Fig. 5B,C). Liu et al. found that interleukin-1 receptor antagonist fused with serum albumin was selectively delivered to inflamed joints^20^. Coppieters et al. reported that anti-mouse TNF-α VHH fused with anti-HSA VHH more effectively accumulated in inflamed tissues than anti-mouse TNF-α VHH that did not fuse with anti-HSA VHH^21^. It has been suggested that serum albumin-specific transporters and receptors are involved in HSA-based drug delivery to inflamed tissues^32^. A previous study reported that SPARC (secreted protein acidic and rich in cysteine) was overexpressed in inflamed synovial fluid and promoted the accumulation of HSA-based nanomedicines in inflamed joints^33^. Thus, it is possible that the binding ability of the anti-HSA NANOBODY® molecule to MSA increased the distribution of ozoralizumab under inflammatory conditions, but further investigation is needed.

Our study had two limitations. First, the half-life of ozoralizumab differs in humans and rodents. The half-life of ozoralizumab, consisting of an anti-HSA NANOBODY® molecule, is extended by targeting endogenous serum albumin, and depends on the half-life of the serum albumin of the injected species^21,34^. The half-life of ozoralizumab in the serum is approximately three weeks in humans, while it was approximately 24 h in the rodents in the present study, which is in good agreement with the difference in the half-life of HSA and MSA^35–37^. Although these differences in the half-life of ozoralizumab between humans and mice can affect the elimination rate, the present study focused mainly on the absorption and distribution processes within one or two days of injection. Hence, the impact of the shorter half-life would be limited.

Second, adalimumab binds to mouse TNF-α, whereas ozoralizumab does not. Previous studies reported that the TNF-α concentrations in CIA mice were several hundreds of pg/mL^38,39^. On the other hand, the adalimumab concentrations in the CIA mice were around several μg/mL, which suggests the TNF-α concentrations were at least lower than one percent of the adalimumab concentrations throughout the current study period. In addition, TNF-α is predominantly a soluble target. Taken together, target binding may have no or little influence on the PK of adalimumab.

In conclusion, we compared the biodistribution kinetics of subcutaneously injected ozoralizumab and adalimumab, and demonstrated that ozoralizumab was absorbed from the subcutaneous interstitium and transferred to the joint tissues more rapidly than adalimumab in both naive and CIA mice. Our investigation provides new insights for drug selection in clinical practice.

## Methods

### Fluorescence labeling of ozoralizumab and adalimumab

Ozoralizumab was generated at Ablynx by a previously described method^21^. Adalimumab was purchased from Eisai Co., Ltd (Tokyo, Japan). Ozoralizumab and adalimumab were de-salted on a PD-10 column (GE Healthcare, 17-0851-01, IL, USA) to remove amine-containing reagents, and labeled with Alexa Fluor 680 (SAIVI Rapid Antibody Labeling Kit, Invitrogen, S30045, MA, USA), in accordance with the manufacturer's instructions.

### Animals

Male DBA/1 J mice aged 7–8 weeks old were purchased from Japan SLC (Shizuoka, Japan). All the mice were housed in a temperature- and humidity-controlled facility maintained under a 12-h light/dark cycle, and had free access to standard food and water. All of our experimental protocols were approved by the Animal Experimental Committee of Osaka University (approval number; 01-071-002). All methods were carried out following relevant guidelines and regulations, and the study was carried out in compliance with the ARRIVE guidelines.

### Induction of CIA

CIA was induced according to the protocol recommended by Japan SLC. In brief, bovine type II collagen solution (8 mg/mL, 0.01 M phosphate acetate buffer, Collagen Research Center, Tokyo, Japan) was mixed with an equal volume of Freund's incomplete adjuvant (BD, NJ, USA) containing 4 mg/mL *Mycobacterium tuberculosis* H37Ra (BD, NJ, USA), to obtain a 4 mg/mL type II collagen emulsion. Then, the mice were injected intradermally at the base of the auricle with 25 μL of the emulsion. At three weeks after the first immunization, the mice were injected intradermally at the base of the tail with 25 μL of the emulsion.

### Assessment of arthritis score

Macroscopic examinations of CIA mice for signs of arthritis were performed at five weeks after the first immunization, as previously described^40^, on a scale of 0 to 4: 0 = normal paw; 1 = only one toe inflamed and swollen; 2 = more than one toe, but not entire paw, inflamed and swollen, or mild swelling of entire paw; 3 = entire paw inflamed and swollen; 4 = entire paw very inflamed and swollen. Mice with low-level arthritis score were excluded from the experiments. The paws of CIA mice with arthritis score of 0–4 were sectioned in the sagittal plane and stained with hematoxylin & eosin (HE) to confirm the histopathological findings associated with each arthritis score. Edema of the subcutaneous tissues and inflammatory cell infiltration became more and more marked as the arthritis score increased (Supplementary Fig. 2).

### In vivo biofluorescence imaging and analysis

At five weeks after the first immunization with the type II collagen emulsion, the arthritis scores were assessed and 12 CIA mice were assigned at a ratio of 1:1 to the Alexa 680-labeled ozoralizumab (ozoralizumab-Alexa 680) group (n = 6) and Alexa 680-labeled adalimumab (adalimumab-Alexa 680) group (n = 6), with comparable body weights and arthritis scores between the two groups. Twelve age-matched naive mice were also divided into two groups (n = 6), with comparable body weights between the two groups. The day after arthritis score evaluation and assignment, the CIA mice and naive mice received a single subcutaneous injection of 2 mg/kg ozoralizumab-Alexa 680 or adalimumab-Alexa 680, as previously described^41^. Before, and at 4, 8, 24, 48, and 72 h after the injection, the mice were anaesthetized with 2–3% isoflurane (Pfizer, NY, USA) and fluorescence photographs were obtained from the dorsal side, using the IVIS Lumina II Imaging System (CaliperLS, MA, USA), at an excitation wavelength range of 615–665 nm and emission wavelength range was 695–770 nm. The parameters were set by reference to a previous report, with some modifications, as follows: exposure time = 3 s; f-stop = 2; binning factor = 4; field of view = 12.5^41^. The acquired images were analyzed using Living Image® software version 4.2. Each fore/hind paw was surrounded by a circle on the images as the region of interest (ROI), and the average radiant efficiency (p/sec/cm^2^/sr)/(μW/cm^2^) was measured in the ROI. The value obtained for each paw was treated as separate data. Area Under Curve (AUC) determination for the fluorescence data were analyzed using the PKNCA R package (version 0.9.4). To calculate the ratio in the CIA to naive animals, the radiant efficiency in each paw of the CIA mice was divided by the mean of average radiant efficiency for all paws in the naive mice at each time-point.

### Fluorescence microscopy, immunohistochemistry, and morphometry

At five weeks after the first immunization, the arthritis scores were assessed and nine CIA mice were assigned to the saline, ozoralizumab-Alexa 680, or adalimumab-Alexa 680 groups (n = 3 each) with comparable body weights and arthritis scores. In the same manner as for the in vivo biofluorescence imaging, the day after arthritis score evaluation and assignment, the mice received a single subcutaneous injection of 2 mg/kg ozoralizumab-Alexa 680 or adalimumab-Alexa 680. At four and eight hours after the injection, the mice were sacrificed by exsanguination through the abdominal aorta under deep isoflurane anesthesia and then necropsied to collect the paws. The paws were trimmed into sagittal sections, frozen in SCEM compound (SECTION-LAB Co. Ltd., Hiroshima, Japan), and sectioned at 10 μm using Cryofilm (SECTION-LAB Co. Ltd.), according to the Kawamoto film method^42^. Unstained sections were prepared to evaluate the distribution of fluorescence of Alexa 680 and examined by microscopy (BX-710; KEYENCE Co., Osaka, Japan). Sections were also stained with hematoxylin & eosin (HE) to identify the tissues. Immunohistochemistry was performed in the CIA mice at eight hours after the injections, using anti-mouse serum albumin rabbit polyclonal antibody (5 μg/mL; Proteintech Group Inc., IL, USA) or control rabbit polyclonal antibody (5 μg/mL; Novus Biologicals LLC., CO, USA). After incubation with Alexa 488-labeled anti-rabbit Fab antibody (Jackson ImmunoResearch Inc., PA, USA), the sections were coverslipped for microscopic examination and morphometry. For analysis of the histological images of the unstained sections and immunohistochemistry sections of the talocrural joints in the CIA mice at eight hours after the subcutaneous injections, the fluorescence intensity of Alexa 680 in the unstained sections and that of Alexa 680 merged with the fluorescence of Alexa 488-label bound to anti-mouse albumin antibody in the immunohistochemistry sections were measured in the joint cavity, and the fluorescence intensities per unit area of the joint cavity was calculated.

### Pharmacokinetic experiment

At five weeks after the first immunization, the arthritis scores were assessed and eight CIA mice were assigned to the ozoralizumab-Alexa 680 group (n = 4) or adalimumab-Alexa 680 group (n = 4) with comparable body weights and arthritis scores. Age-matched naive mice were also divided into the same two groups. The day after arthritis score evaluation and assignment, each mouse received a subcutaneous injection of 2 mg/kg ozoralizumab-Alexa 680 or adalimumab-Alexa 680 in the back. At 1, 2, 4, 6, 8, 24, 48, and 72 h after the injection, 40–50 µL of blood was collected at each time-point from the tail vein. The blood was centrifuged (11,200 × *g*, 4 °C, 6 min) to separate the serum. The antibody concentrations in the serum were determined using a fluorescence microplate reader (Infinite 200, Tecan Group Ltd.) at an excitation wavelength of 640 nm and emission wavelength of 702 nm. The serum concentration–time profiles of ozoralizumab-Alexa 680 and adalimumab-Alexa 680 were analyzed using Phoenix WinNonlin 6.2 (Certara) by 1-compartmental analysis for the absorption rate constant (k_a_), and by non-compartmental analysis for the maximum concentration (C_max_), time-to-maximum concentration (t_max_), area under the serum concentration–time curve from 0 to 72 h (AUC_0–72 h_), and the elimination half-life (t_1/2_).

### Statistical analysis

For analyzing the results of the biofluorescence imaging, the statistical significance of differences between two groups was determined by Welch’s t-test, and the statistical significance of differences among multiple groups was determined by two-way analysis of variance (ANOVA), followed by Tukey’s post hoc test. In regard to analysis of the results of fluorescence microscopy and immunohistochemistry, the statistical significance of differences between the two groups was determined by the Wilcoxon rank sum test. For the pharmacokinetic analysis, the statistical significance of differences between the two groups was determined by Welch’s t-test. A significance level of 95% with a p value of 0.05 was used for all the statistical tests. R version 4.1.1. software was used for all the statistical analyses.

## Supplementary Information

Supplementary Information 1.

Supplementary Information 2.

Supplementary Information 3.

## Data Availability

The datasets used and/or analyzed in the current study are available from the corresponding author on reasonable request.
