# Supplementary figures and images for: A novel anti-TNF-α drug ozoralizumab rapidly distributes to inflamed joint tissues in a mouse model of collagen induced arthritis
Source: Sci Rep. 2022 Oct 27;12:18102. doi: 10.1038/s41598-022-23152-6 (PMC9613905; doi:10.1038/s41598-022-23152-6)

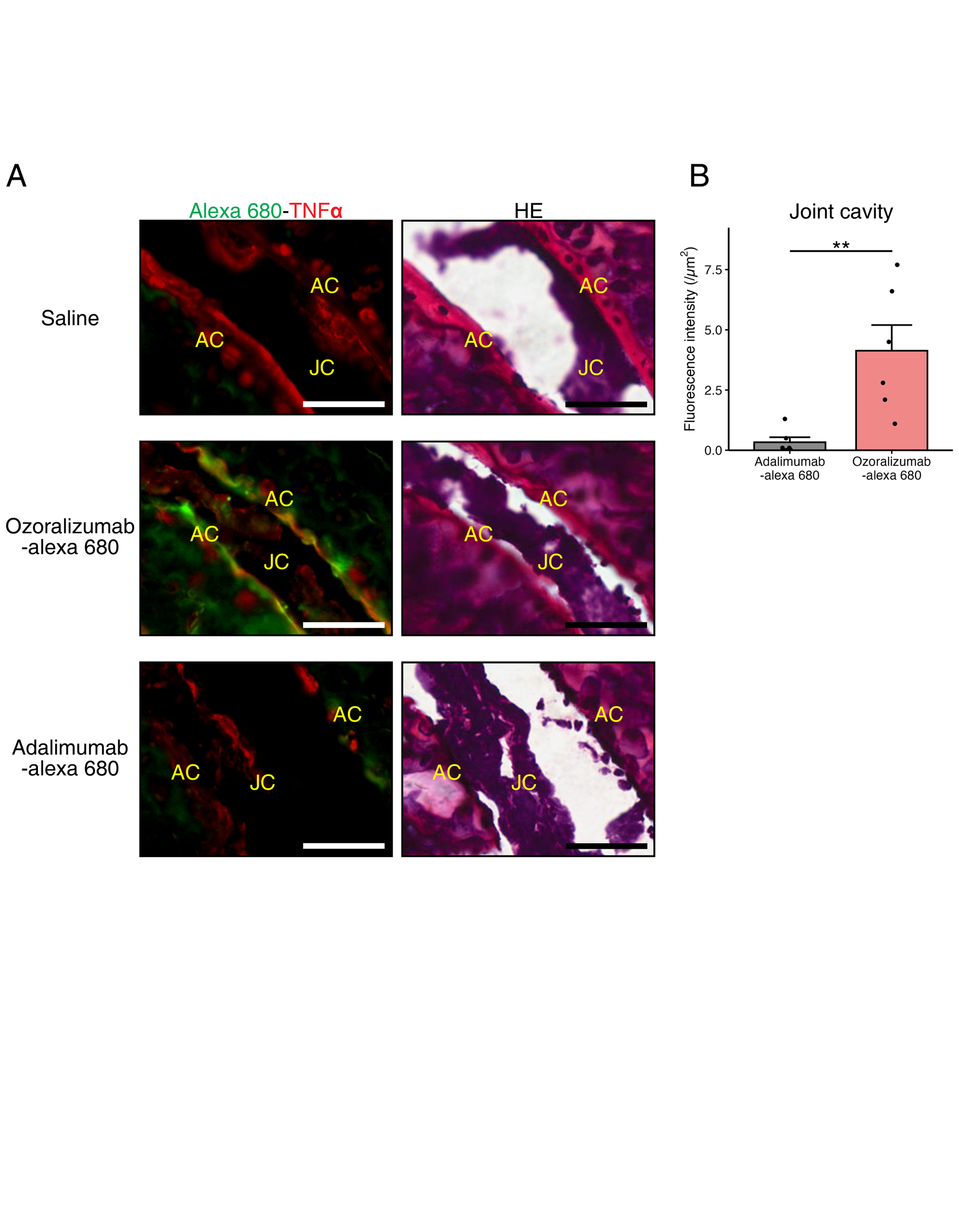

Supplement: Supplementary file 1 — Supplementary Information 1. [file 41598_2022_23152_MOESM1_ESM.tif]

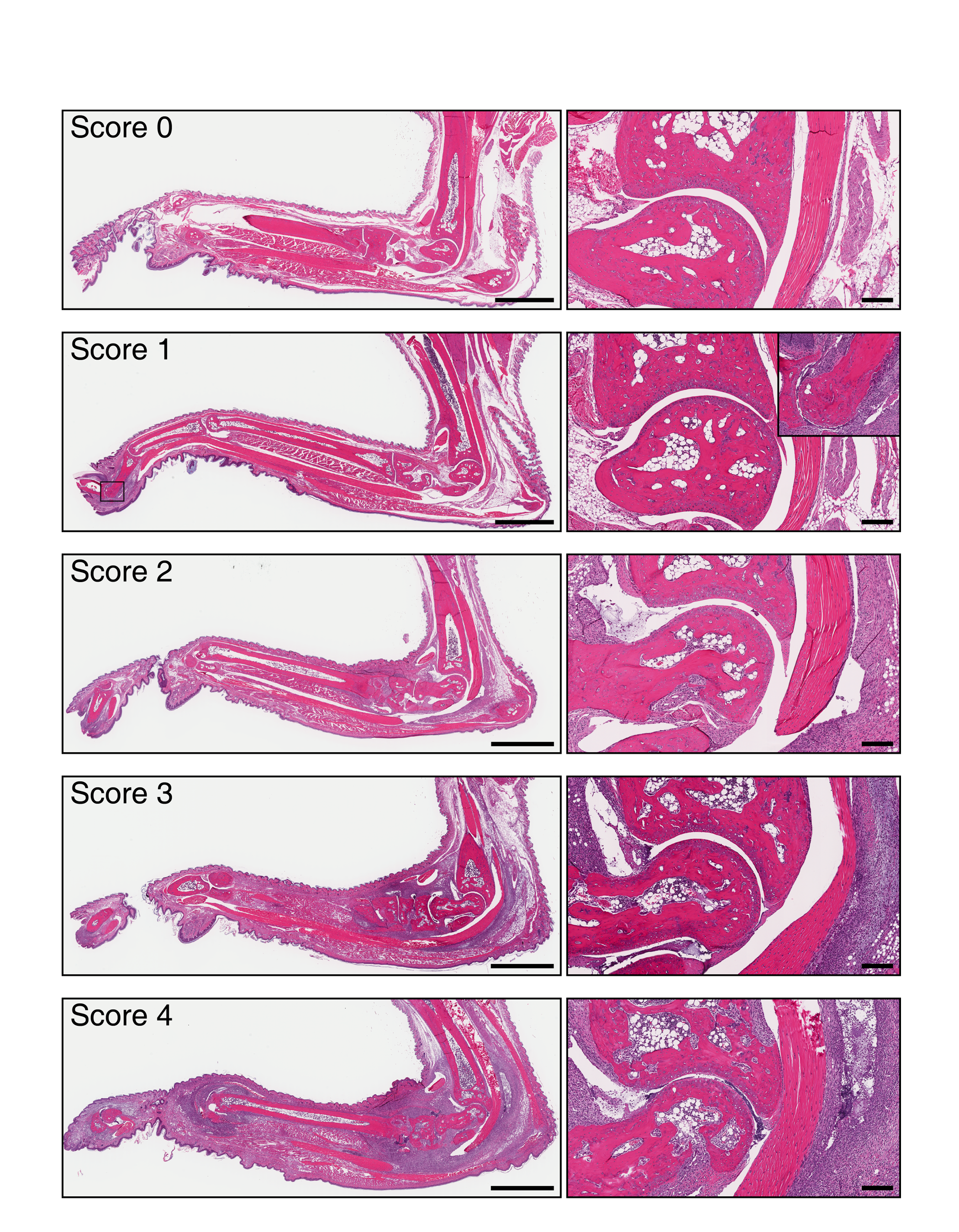

Supplement: Supplementary file 2 — Supplementary Information 2. [file 41598_2022_23152_MOESM2_ESM.tif]
