# Supplementary material for: A novel anti-TNF-α drug ozoralizumab rapidly distributes to inflamed joint tissues in a mouse model of collagen induced arthritis
Source: Sci Rep. 2022 Oct 27;12:18102. doi: 10.1038/s41598-022-23152-6 (PMC9613905; doi:10.1038/s41598-022-23152-6)
Supplement: Supplementary file 3 — Supplementary Information 3. [file 41598_2022_23152_MOESM3_ESM.docx]

**Supplementary Material and Methods**

Measurements of the binding affinity of ozoralizumab for albumin

Binding kinetics between ozoralizumab and mouse or human serum albumin were measured by the Surface Plasmon Resonance (SPR) technique (Biacore 4000, cytiva, Tokyo, Japan) using the Biacore 4000 instrument, in accordance with the instruction manual. Briefly, mouse serum albumin or human serum albumin was immobilized onto the Series S Sensor Chip CM5 using amine coupling. Ozoralizumab and ozoralizumab-Alexa 680 were individually coupled onto the sensor chip for an association time of 90 seconds, followed by dissociation kinetics at a dissociation time of 600 seconds. The association rate constant (k_a_) and dissociation rate constant (k_d_) were calculated by nonlinear regression analysis of the primary sensorgram according to a 1:1 binding model. The dissociation constant (K_d_) was calculated using the formula, K_d_ = k_off_/k_on_.

Immunohistochemistry with the TNFα antibody

Immunohistochemistry was performed on the talocrural joints of mice sacrificed at eight hours after injection, using anti-mouse TNFα rabbit polyclonal antibody (5 μg/mL; Bioss Inc., MA, USA) or control rabbit polyclonal antibody (5 μg/mL; Novus Biologicals LLC., CO, USA). After incubation with Alexa 488-labeled anti-rabbit Fab antibody (Jackson ImmunoResearch Inc., PA, USA), the sections were coverslipped for microscopic examination and morphometry. Histological images of immunohistochemistry sections of the talocrural joints of the CIA mice at eight hours after subcutaneous injection of the drug were captured with the BX-710 microscope (KEYENCE Co.). For the joint cavity, the fluorescence values of Alexa 680 merged with the fluorescences of Alexa 488-label bound to anti-mouse TNFα antibody in the immunohistochemistry sections were measured, and the fluorescence values per unit area of the joint cavity were calculated.

**Supplementary table 1. The binding affinity of ozoralizumab for serum albumin.**

| Samples | Ligands | k_a_ (1/Ms) | k_d_ (1/s) | K_d_ (M) |
| --- | --- | --- | --- | --- |
| Ozoralizumab | HSA | 3.1 x 10^5^ | 5.5 x 10^-3^ | 1.8 x 10^-8^ |
|  | MSA | 2.5 x 10^5^ | 9.9 x 10^-2^ | 4.0 x 10^-7^ |
| Ozoralizumab-  Alexa 680 | HSA | 1.2 x 10^5^ | 5.1 x 10^-3^ | 4.3 x 10^-8^ |
|  | MSA | 9.8 x 10^4^ | 9.3 x 10^-2^ | 9.5 x 10^-7^ |

The binding affinity of ozoralizumab for serum albumin was assessed using Surface Plasmon Resonance (SPR). HSA indicates human serum albumin, MSA indicates mouse serum albumin, k_a_ indicates the association rate constant, k_d_ indicates the dissociation rate constant, K_d_ indicates the dissociation constant. Values are geometric means of 3 replications.

**Supplementary Table 2. Pharmacokinetic parameters in the serum of Alexa 680-labeled ozoralizumab (ozoralizumab-Alexa 680) and Alexa 680-labeled adalimumab (adalimumab-Alexa 680) after a single subcutaneous injection at 2 mg/kg in mice.**

| Antibody | Animal  model | C_max_  (μg/mL) | t_max_  (h) | t_1/2_  (h) | AUC_0-72h_  (h*μg/mL) | k_a_  (h^-1^) |
| --- | --- | --- | --- | --- | --- | --- |
| Ozoralizumab-  Alexa 680 | Naive | 6.91 ± 0.57 | 16 ± 9 | 23 ± 4 | 311 ± 19 | 0.123 ± 0.051 |
|  | CIA | 6.14 ± 0.54 | 8.0 ± 0.0 | 23 ± 3 | 251 ± 19 | 0.154 ± 0.016 |
| Adalimumab-  Alexa 680 | Naive | 12.2 ± 1.9 | 30 ± 12 | 92 ± 28^a^ | 660 ± 84 | 0.0395 ± 0.0200 |
|  | CIA | 7.75 ± 1.35 | 24 ± 0 | 110 ± 40 | 453 ± 80 | 0.0561 ± 0.0155 |

Each value represents the mean ± S.D. of four mice.

a: The value represents the mean ± S.D. of three mice, except for one mouse in which the value could not be calculated.

C_max_ indicates the maximum concentration reached after the injection. T_max_ indicates the time at which the maximum concentration was reached. T_1⁄2_ indicates the elimination phase half-life. AUC indicates the area under the curve. k_a_ indicates the absorption rate constant. S.D.: Standard deviation.

**Supplementary figure 1. Localization of mouse TNFα and test articles in the talocrural joints.**

1. At eight hours after a single subcutaneous injection of ozoralizumab-Alexa 680 or adalimumab-Alexa 680, immunohistochemistry was performed on sagittal sections of the talocrural joints with the antibody against mouse TNFα. The green fluorescence represents the administered compounds labeled with Alexa 680 and the red fluorescence represents mouse TNFα. Serial sections were stained with HE (right). Scale bar: 50 µm. AC: articular cartilage; JC: joint cavity.
2. The fluorescence values of Alexa 680 merged with the fluorescences of Alexa 488-labeled TNFα antibody in immunohistochemistry sections of the joint cavity at eight hours after subcutaneous injection of the drug were analyzed. Dot plots represent each individual data and bars represent the means ± SEM. *P* values were calculated using the Wilcoxon rank sum test. ***p* < 0.01.

**Supplementary figure 2. Arthritis score and histological examination.**

CIA mice paws (arthritis score = 0–4) were sectioned in the sagittal plane and stained with hematoxylin & eosin (HE) to confirm the histopathological findings associated with each arthritis score. Edema of the subcutaneous tissues and inflammatory cell infiltration became more and more marked as the arthritis score increased. Scale bar: 2 mm (left), 200 µm (right).
